# Progenitor Cell Therapy for the Treatment of Central Nervous System Injury: A Review of the State of Current Clinical Trials

**DOI:** 10.4061/2010/369578

**Published:** 2010-07-20

**Authors:** Peter A. Walker, Matthew T. Harting, Shinil K. Shah, Mary-Clare Day, Ramy El Khoury, Sean I. Savitz, James Baumgartner, Charles S. Cox

**Affiliations:** ^1^Department of Surgery, Medical School at Houston, University of Texas, 6431 Fannin Street, MSB 5.236, Houston, TX 77030, USA; ^2^Department of Pediatric Surgery, Medical School at Houston, University of Texas, 6431 Fannin Street, MSB 5.236, Houston, TX 77030, USA; ^3^Department of General Surgery, Medical School at Ann Arbor, University of Michigan, Ann Arbor, MI 48109, USA; ^4^Michael E DeBakey Institute for Comparative Cardiovascular Science and Biomedical Devices, Texas A & M University, College Station, TX 77843, USA; ^5^Department of Neurology, Medical School at Houston, University of Texas, Houston, TX 77030, USA; ^6^Department of Pediatric Neurosurgery, Medical School at Houston, University of Texas, Houston, TX 77030, USA

## Abstract

Recent preclinical work investigating the role of progenitor cell therapies for central nervous system (CNS) injuries has shown potential neuroprotection in the setting of traumatic brain injury (TBI), spinal cord injury (SCI), and ischemic stroke. Mechanisms currently under investigation include engraftment and transdifferentiation, modulation of the locoregional inflammatory milieu, and modulation of the systemic immunologic/inflammatory response. While the exact mechanism of action remains controversial, the growing amount of preclinical data demonstrating the potential benefit associated with progenitor cell therapy for neurological injury warrants the development of well-controlled clinical trials to investigate therapeutic safety and efficacy. In this paper, we review the currently active or recently completed clinical trials investigating the safety and potential efficacy of bone marrow-derived progenitor cell therapies for the treatment of TBI, SCI, and ischemic stroke. Our review of the literature shows that while the preliminary clinical trials reviewed in this paper offer novel data supporting the potential efficacy of stem/progenitor cell therapies for CNS injury, a great deal of additional work is needed to ensure the safety, efficacy, and mechanisms of progenitor cell therapy prior to widespread clinical trials.

## 1. Introduction

Central nervous system (CNS) insults including ischemic stroke, traumatic brain injury (TBI), and traumatic spinal cord injury (SCI) represent a major burden to the healthcare system worldwide. Approximately 5 million people are burdened by the long-term physical, cognitive, and psychosocial deficits associated with TBI in the United States with 40% of patients reporting unmet needs 1 year post injury [1]. Furthermore, a recent analysis of stroke patients has shown a lifelong health burden of 9.5 quality adjusted life years associated with an initial cerebrovascular accident [2]. Overall, the economic impact of traumatic and acute CNS insults reaches several billion dollars in the United States alone [3, 4].

Preliminary research is currently underway to evaluate the potential efficacy of adult tissue progenitor (stem) cell therapies for the treatment of CNS injury. Adult tissue progenitor cells are located in select microenvironments (niches) which protect against overproliferation and depletion as well as regulate progenitor cell involvement in resident tissue repair and regeneration [5]. By definition, progenitor cells are multipotent with the capacity of self-renewal [6] making them prime candidates for the development of novel therapies. Our paper will focus on progenitor cell populations derived from the bone marrow and umbilical cord blood niches including mesenchymal stromal cells (MSCs) and hematopoietic stem cells (HSCs). There are currently no clinical trials utilizing embryonic stem cells or induced pluripotent stem cells for TBI, stroke, or SCI.

## 2. Therapeutic Mechanism

Recent preclinical work investigating bone marrow derived-progenitor cell therapies for CNS injury has shown potential neuroprotection after TBI [7], ischemic stroke [8], and SCI [9]. While initial research indicated engraftment and transdifferentiation into neural cells could explain the observed benefit [10], the exact mechanism remains controversial. Potential mechanisms currently under investigation include engraftment and transdifferentiation, modulation of the locoregional inflammatory milieu, and modulation of the systemic immunologic/inflammatory response.

### 2.1. Engraftment and Transdifferentiation

Preliminary research completed in the Chopp laboratory showed both motor and cognitive improvement after the intravenous injection of MSCs in a rodent TBI model. The injected MSCs were found to migrate towards the site of injury, engraft, and display neuronal (neuronal nuclei (NeuN)) and astrocytic (glial fibrillary acidic protein (GFAP)) markers [11]. Hayase et al. have successfully induced MSCs to form neural spheres *in vitro *with subsequent implantation into rodent cortex after focal ischemic injury. The implanted progenitor cells were found to display neural cell markers and engraft up to 100 days after injury with associated behavioral recovery [12]. The Ha laboratory implanted umbilical cord blood-derived progenitor cells (HUCBCs) into the injury site after spinal cord contusion in a rodent model. The HUCBCs were found to engraft at the site of injury and differentiate into neural cells as evident by GFAP and microtubule-associated protein 2 (MAP2) staining. Locomotor testing showed functional improvement for all time points tested up to 8 weeks after SCI [13].

Despite the promising research showing engraftment and transdifferentiation of transplanted progenitor cells into neural cells, the importance of engraftment and frequency of transdifferentiation remain extremely controversial [14, 15]. The implantation of HSCs into murine striatum [16] and injury zone of spinal cord contusion [17] showed differentiation into macrophages or microglia but failure to transdifferentiate into neurons. Furthermore, the implantation of MSCs into demyelinated spinal cord showed migration into normal tissue and failure of transdifferentiation associated with collagen deposition and further axonal injury [18]. While the direct implantation of progenitor cells with transdifferentiation into neurons could afford neuroprotection, the low frequency of engraftment and differentiation may limit this pathway as a mechanism for functional recovery.

### 2.2. Modulation of the Locoregional Inflammatory Response

Progenitor cell migration towards the site of injury and interaction with resident microglia could modulate the locoregional inflammatory response leading to enhanced neuroprotection. Harting et al. investigated the local intracerebral inflammatory response after TBI by completing a series of rodent cortical injuries followed by the harvest of intracerebral fluid from 6 to 72 hours after injury. Multiplex cytokine analysis of the intracerebral fluid showed an increase in the proinflammatory cytokines IL-1*α*, IL-1*β*, IL-6, and TNF*α* in the direct injury and penumbral areas [19]. The observed increase in cytokine production offers an attractive target for novel cell therapies.

Coculture of human MSCs with immunologic cells (dendritic cells and T cells) was associated with an increase in the anti-inflammatory cytokines interleukin 4 (IL-4) and interleukin 10 (IL-10) in accordance with a decrease in the proinflammatory cytokine interferon gamma (IFN-*γ*). An increase in the proportion of T regulatory cells with MSC coculture was also observed [20]. Walker et al. directly implanted MSCs into the cortex of rodents after TBI and found an increase of interleukin 6 (IL-6) in brain tissue supernatants. Subsequently, a series of *in vitro *MSC and neuronal stem cell (NSC) cocultures showed activation of the NSC NF*κ*B pathway leading to a decrease in apoptosis [21]. The Wen laboratory has shown increased intracerebral IL-10 with decreased tumor necrosis *α* (TNF*α*) leading to improved cognitive performance after the direct implantation of MSCs using a rodent ischemic stroke model [22]. These promising preliminary studies have shown that modulation of the locoregional postinjury proinflammatory environment could afford neuroprotection.

### 2.3. Modulation of the Systemic Immunologic Response

Recent work completed in the Pennypacker laboratory has shown that the adrenergic output associated with rodent ischemic stroke leads to the release of immunologic cells (T cells) from the spleen into the systemic circulation causing a reduction in splenic mass and an increase in cavity volume. Treatment with the panadrenergic blocker carvediol prevented the loss of splenic mass and decreased cavity volume [23]. Using a similar model, Verdrame et al. have shown that the immunologic cells released into the systemic circulation are mainly composed of cytotoxic CD8+ T cells potentially exacerbating neurological injury observed with ischemic stroke. The intravenous injection of HUCBCs in the stroke model also prevented the loss of splenic mass and decreased cavity size likely via a reduction in CD8+ cell release [24].

Additional research investigating the potential interaction between transplanted progenitor cells and lung immunologic cells is currently underway. Using a murine sepsis model, the Mezey laboratory has shown that the intravenous injection of MSCs is associated with decreased mortality and improved organ function. The observed benefit was derived from interactions between the injected MSCs and lung macrophages leading to increased IL-10 production via a prostaglandin E2-dependent mechanism. Furthermore, the beneficial effect was eliminated by the administration of antibodies to either IL-10 or the IL-10 receptor thereby confirming the importance of anti inflammatory cytokine production in therapeutic efficacy [25].

There is limited data on the interaction of implanted adult progenitor cells with other organ systems in the setting of neurologic injury. Distribution studies have demonstrated localization of implanted cells to liver and kidney in addition to the more commonly described spleen and lung after intravenous or intra-arterial administration [26–29]. To our knowledge, there is no published data on the liver and/or kidney acting as potential bioreactors for cytokine/growth factor secretion after progenitor cell therapy for neurologic injury.

Preliminary preclinical work investigating the potential role of progenitor cell therapeutics for CNS injury has shown promise. The mechanism of the observed benefit remains controversial; however, more recent data questions the frequency and clinical significance of transdifferentiation as well as the volume of cells reaching the injury site due to a significant pulmonary first-pass effect [30]. We believe that a more plausible explanation is that for some types of cell-based therapies, the transplanted cells are interacting with distant organ systems leading to alteration in the systemic inflammatory/immunologic response. Progenitor cells could interact with resident lung macrophages and splenic T cells leading to an increase in anti inflammatory cytokine production. The observed increase in systemic anti inflammatory cytokine concentrations may affect the resident brain microglia accounting for the observed therapeutic benefit.

## 3. Clinical Trials

The growing amount of preclinical data showing the potential benefit associated with progenitor cell therapy warrants the development of well-controlled clinical trials to investigate therapeutic safety and efficacy for central nervous system insults such as ischemic stroke, SCI, and TBI. Below we review the preliminary clinical trials investigating progenitor cell therapy for CNS insults completed to date.

### 3.1. Ischemic Stroke

Stroke is a leading cause of long-term disability in the United States [31]. Intravenous tissue plasminogen activator (tPA) is the only proven treatment for acute ischemic stroke within the first three hours of symptom onset [32]. Cell-based therapies have emerged as a novel and highly promising investigational approach to enhance recovery after stroke in animal models [33–37]. The encouraging preliminary results have led several investigators to launch clinical trials evaluating the safety of cell-based therapies in stroke patients. The safety, feasibility, ideal cell type, optimal dosage, and most favorable delivery method of cells are currently unknown.

Savitz et al. are currently conducting a prospective, Phase 1 trial (http://www.clinicaltrials.gov/ Identifier: NCT00859014) evaluating the safety of bone marrow aspiration and infusion of bone marrow mononuclear cells (BMMCs) in adults within 24–72 hours of ischemic stroke. Primary outcome measures include a series of short- and long-term safety assessments with a secondary evaluation of neurological function as measured up to 90 days after injury. Autologous BMMCs are administered via peripheral intravenous injection followed by serial measurements of hemodynamic variables to assess immediate postinfusion safety. Selected inclusion criteria are middle cerebral artery (MCA) territory infarct, age between 18 and 80 years, and National Institutes of Health Stroke Scale (NIHSS) between 6 and 20. 

Lin et al. (China University Hospital, Taichung, Taiwan) have recently completed a Phase 1 clinical trial ensuring the safety of the direct intracerebral transplantation of CD34+ progenitor cells in stroke patients. The CD34+ progenitor cells were obtained from peripheral blood of patients with MCA strokes occurring within the past 6 to 60 months. Currently, a Phase 2 trial is recruiting patients to determine the potential efficacy of CD34+ cell implantation (http://www.clinicaltrials.gov/ Identifier: NCT00950521). The treatment group is to receive conventional rehabilitation as well as the direct implantation of CD34+ progenitor cells with the control group receiving conventional rehabilitation alone. The primary outcome is NIHSS scores collected serially for up to 12 months. Inclusion criteria are patients between 35 and 70 years old and an NIHSS between 9 and 20. The investigators plan to enroll 60 patients and is currently underway.

Hernández et al. (Hospital Universitario Central de Asturias, Asturias, Spain) are currently enrolling patients for a Phase 2 trial investigating the safety and efficacy of the intra arterial delivery of autologous CD34+ progenitor cells into the MCA after ischemic stroke. Cellular harvest and injection occurs between 5 and 9 days after the onset of stroke symptoms. Patient hemodynamics and neurologic status are monitored in the acute setting with follow up exams up to 6 months after treatment. Adverse events are classified as any worsening of the neurologic exam. Therapeutic efficacy is determined via serial physical, laboratory, and radiographic exams. Selected inclusion criteria are symptoms and signs of clinically definite MCA acute stroke (http://www.clinicaltrials.gov/ Identifier: NCT00761982). 

André et al. (Federal University of Rio de Janeiro, Rio de Janeiro, Brazil) are conducting a Phase 1 clinical trial investigating the intravenous and intra arterial injection of the autologous bone marrow-derived mononuclear cell fraction within 90 days of MCA stroke. A cell dosage of up to 500 × 10^6^ mononuclear cells will be used. Patients will be monitored with serial physical and radiographic exams up to 4 months after treatment. Adverse events will be recorded as any worsening in neurologic exam. Transcranial doppler will be used during intra arterial injection to ensure adequate blood flow in the middle cerebral artery. Improvement in neurologic deficits and neuroimaging will be recorded as secondary outcome measures during the study time period. Patients who are between 18 and 75 years old with an MCA infarct documented on imaging, and NIHSS scores between 4 and 20 are eligible (http://www.clinicaltrials.gov/ Identifier: NCT00473057).

Habib et al. (Imperial College London, London, England) are completing an additional Phase 1 trial investigating the safety of intra arterial injection of the autologous bone marrow CD34+ progenitor cell population into the MCA of patients with acute anterior circulation strokes. Safety is to be assessed by physical exam and laboratory parameters. Improvement in clinical function as assessed by the Modified Rankin Score and NIHSS is secondary outcome that will be evaluated. Selected inclusion criteria include a clinically definite acute stroke with known onset time, ability to start treatment within 7 days of onset, and patients between 30 and 80 years old. Data collection for the trial is set to be completed in May 2010 (http://www.clinicaltrial.gov/ Identifier: NCT00535197).

Preliminary clinical trials investigating the role of cell therapeutics for ischemic stroke have been limited to date and powered only to evaluate safety (Table 1). The majority of these studies are restricting enrollment to patients with MCA infarcts territory and do not assess the role of these cells in other areas of the brain. No optimal method of delivery has been established, and it is unclear whether intravenous, intra-arterial, or other approaches may be safer and lead to better outcomes. Additionally, the studies employ different outcome measures limiting the ability to compare results among trials. While these preliminary studies have yielded some data to support the safety of cellular transplantation, additional trials need to be completed prior to controlled multicenter trials. A recent consensus conference (STAIR) was convened to discuss the future of clinical trials in stroke. The consensus highlighted the need for well-designed clinical trials with cell therapy being an excellent candidate [38].

### 3.2. Traumatic Brain Injury

A search of the Clinicaltrials.gov database identified 279 ongoing or recently completed clinical trials for patients with TBI (search performed 3/31/2010). The treatments were focused on both acute therapy, as well as ongoing or chronic therapy, and included (but were not limited to) medications (i.e., amantadine, carbamazepine, oxycyte, or erythropoietin), hyperbaric oxygen, hypothermia, educational interventions, and physical rehabilitation. Using the search terms “stem cell” and “brain” identified nearly 300 studies, including studies evaluating the use of adult stem cells (administered via various routes, in various numbers, and using various cell types) to treat hypoxic/ischemic encephalopathy, cerebral palsy, multiple sclerosis, amyotrophic lateral sclerosis, neuronal ceroid lipofuscinosis, Parkinson's disease, and others.

A single Phase I study using bone marrow-derived mononuclear cells in children after isolated TBI has recently been completed. In this study, 10 children age 5–14 years with a Glasgow coma scale score of 5–8 were treated with 6 × 10^6^ bone marrow-derived mononuclear cells per kg body weight delivered intravenously within 48 hours of an isolated TBI. To determine the safety of administration, systemic and cerebral hemodynamics, laboratory parameters, chest radiographs, and serial clinical assessments were monitored. Additionally, serial cerebral magnetic resonance imaging, neuropsychologic evaluation, and functional outcome measures were obtained as preliminary measures of efficacy.

There were no identifiable adverse events with close monitoring of the neurologic, pulmonary, renal, hepatic, and hematologic systems. Functional and neuropsychological testing, including the Glasgow Outcome Scale, the Pediatric Injury Functional Outcome Scale, and the Wechsler Abbreviated Scale of Intelligence, revealed recovery consistent with (or improved from) expected baselines. Magnetic resonance imaging volumetric data revealed no significant change in grey matter, white matter, intracranial volume, or CSF space at 1 and 6 months as measured relative to expected norms [39].

This study should open the door for translation of cell therapies, particularly among patients with neurologic diseases and among pediatric patients. Given the apparent safety of this study, the development of larger, multicenter studies to further assess dosing and efficacy of autologous cell therapy for TBI is underway. Additionally, similar (more dispensable) progenitor cell populations, such as cord blood cells, may be safe and efficacious as well and warrant further study.

### 3.3. Spinal Cord Injury

#### 3.3.1. Human Trials Using Autologous BMMC Delivered by Intravenous or Intra-Arterial Infusion

A trial comparing autologous BMMC intravenous transplantation plus physical therapy to physical therapy only in patients with chronic SCI has been reported from Al-Azhar University Military Medical Academy in Cairo, Egypt. No outcomes data has been published on this trial.

Syková et al. treated two groups of patients with autologous BMMC via either intravenous infusion or intra-arterial infusion through the vertebral artery. In the first group (subacute group), cell therapy was delivered between 10 and 33 days following SCI. The second group (chronic group) was treated between 2 and 18 months after SCI. Of the eight subacute patients, four were treated intravenously and four via an intra-arterial route. Two of the chronic patients were treated intra-arterially and the remaining ten patients were treated intravenously. Patients were evaluated 3, 6, and 12 months post BMMC treatment. All four of the subacute patients treated via the intra-arterial route and one of the four treated intravenously experienced improvement in the American Spinal Injury Association (ASIA) score. One of two chronic patients treated via an intra-arterial route experienced an improvement in ASIA score, but none of the remaining 10 chronic patients treated with intravenous administration of BMMCs improved [40].

Cristante et al. treated 39 patients with chronic SCI using peripheral stem cell mobilization (granulocyte monocyte colony stimulating factor (GM-CSF) treatment) and subsequent collection by apheresis. The patients all had complete SCI of two or more years duration. At least 2.5 × 10^6^ CD34+ cells per kg body weight were collected. Cells were delivered intra-arterially into the anterior spinal artery at or near the level of their SCI. Patients were followed with serial somatosensory-evoked potential (SSEP) testing over 30 months. Overall 66.7% of patients experienced improved latency on SSEP evaluation. No difference in response rates was identified between paraplegic or quadriplegic patients [41].

Our group has recently obtained an investigational new drug (IND) application to treat chronic (greater than 6 months post injury) pediatric SCI with autologous BMMC via intravenous infusion. Patients will receive pre- and serial posttreatment neurologic examinations, ASIA Scale ratings, SSEP testing, cystometrogram (CMG) testing, and spinal magnetic resonance imaging with diffusion tensor tractography. We expect to begin enrolling patients by late summer 2010.

#### 3.3.2. Human Trials Using Autologous BMMC Delivered by Lumbar Puncture

Callera et al. treated a total of 10 patients with established SCI. Patients were pretreated with GM-CSF for 5 days and then treated with 100 × 10^6^ autologous BMMC by lumbar puncture (LP). Patients underwent repeat LP 7 days posttreatment and the repeat LPs were reported to be normal. No mention of functional outcome was reported [42]. The same group of investigators treated 16 cases of chronic SCI with either autologous bone marrow-derived CD45+ cells labeled with iron nanoparticles (10 patients) or iron nanoparticles only (6 patients) by lumbar puncture. Serial magnetic resonance imaging scans performed following treatment demonstrated cell migration to the edges of the SCI in 5 of the cell-treated patients but none of the nano-particles only treated patients [43].

#### 3.3.3. Human Trials Using BMMC Delivered by Direct Injection or Surgical Implantation into the Injured Spinal Cord

Deda et al. treated 9 patients with complete SCI (ASIA Grade A) using a processed autologous bone marrow preparation (cells harvested in Turkey, shipped to Ann Arbor, Michigan for processing, and returned to Turkey for cell infusion). Cells were delivered by intravenous infusion, direct injection into the spinal cord above and below the injury site, and by a cell-infused matrix implanted surgically into the injury site. The authors reported improvement to ASIA Grade B of C in treated patients, improved SSEP latencies in treated patients and no adverse events [44].

Yoon et al. treated a total of 35 patients with SCI using direct injection of BMMCs into six sites surrounding the spinal cord injury. Patients were divided into acute (treated within 2 weeks of injury), subacute (treated between 2 and 8 weeks from injury), and chronic (treated greater than 8 weeks from injury) treatment groups. All treated patients also received GM-CSF treatment for 5 months after treatment. A control group of 13 patients who underwent surgery without BMMC or GM-CSF treatment was included in the study. Neurologic improvement (ASIA A to ASIA B or C) was reported in roughly 30% of the acute and subacute treatment groups, but not in in the chronic treatment control groups. Neurologic improvements were greater in patients with the greatest leukocytosis following GM-CSF treatment. Neuropathic pain occurred in a third of the subacute and chronically treated patients but in only one of sixteen acutely treated patients. One control group patient developed neuropathic pain [45].

#### 3.3.4. Human Trials Using Embryonically Derived Stem Cell Products

**Figure 1:**
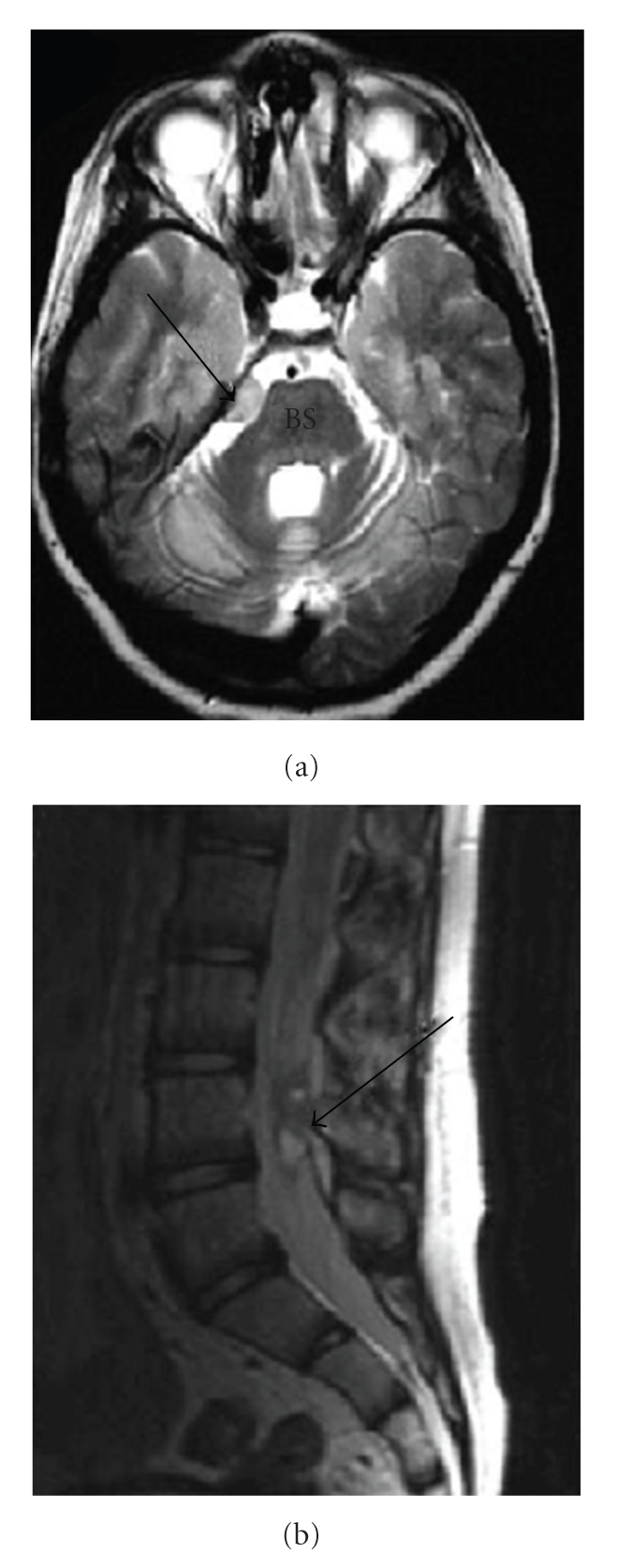
(a) Brain MRI demonstrating a lesion (arrow) based on the tentorium next to the brain stem (BS). (b) Spinal-lumbar MRI (T2) showing an intradural lesion (arrow) at the level of the L4 vertebra. Reproduced with permission.

Considerable regulatory caution has been exercised when human trials using embryonically or fetally derived stem cell products are proposed. Although these more immature cell types have the theoretical advantage of pluripotency, they have also been associated with tumor formation. A case report from Israel describing the development of multifocal CNS glioneuronal tumors following treatment of a child with ataxia telangiectasia using fetal neural stem cells (obtained from multiple human fetuses) has caused researchers and regulators to move cautiously in this area. The tumors were shown to have developed from the transplanted fetal tissue (Figure 1) [46].

Geron Corporation (Menlo Park, Calif, USA) has started a United States Food and Drug Administration (FDA) approved trial using embryonically derived oligodendroglial precursor cells (identical to those used by Kierstadt) to replace myelin-forming cells within injured spinal cords. The cell preparations are injected directly into the spinal cords at the lesion site. The treatment population is restricted to adults with complete thoracic (T3–T9) level SCI. Patients must undergo treatment within 7 to14 days following injury. Preclinical data showing that SCI animals treated with Geron's cell line developed cysts at the level of treatment caused the FDA to put a hold on the clinical trial. Geron and the FDA have reached an agreement to allow the trail to move forward if subsequent preclinical studies provide satisfactory outcome.

## 4. Conclusions

Prior to large, multicenter clinical trials investigating the potential efficacy of progenitor cell therapies for CNS insults, a number of issues need to be addressed. Further research into optimal cell dosing, cell delivery method, and techniques for *in vivo *cell tracking need to be completed to ensure the safety of potential trials while affording them the best possible chance at success. Additional preclinical work to more clearly delineate the progenitor cell mechanism of action would also aid in the planning of quality-controlled clinical studies. Overall, while the very preliminary clinical trials reviewed in this paper offer novel data supporting the potential efficacy of cell therapeutics for CNS injury, a great deal of additional work is needed to ensure the safety and efficacy of progenitor cell therapy prior to widespread clinical trials.

## Figures and Tables

**Table 1:** Listing of location and details of current clinical trials being completed to investigate the potential role of bone marrow-derived progenitor cell therapeutics for the treatment of ischemic stroke.

Location of Study	Study Design	Deliver Route	Sample Size	Cell Type	Inclusion Criteria	Outcomes	Time Window
United States (The University of Texas in Houston)	Single arm	IV	10	Autologous BMMCs	- MCA stroke - 18–80 yo - NIHSS 6 to 20	Safety and feasibility	24 to 72 hrs

Taiwan (The China Medical University Hospital)	Randomized (cell infusion versus conventional treatment)	IC	30	Autologous peripheral blood CD34+ cells	- Stable deficits hemiplegia - 35–70 yo - NIHSS 9 to 20	Safety and efficacy	6 months to 5 years

Spain (Hospital Universitario Central de Asturias)	Single arm	IA	20	Autologous CD34+ bone marrow cells	- MCA stroke - 18–80 yo - NIHSS ≥ 8	Safety	5 to 9 days

France (University Hospital of Grenoble)	Randomized (Control versus 2 treatment groups)	IV	30	Autologous bone marrow derived progenitor cells	- Carotid territory stroke - 18–65 yo - NIHSS > 2	Feasibility and tolerability	6 weeks

United Kingdom (Imperial College London)	Single arm	IA	10	Autologous CD34+ bone marrow cells	- MCA stroke - 30–80 yo - Severe stroke conforming to the TACS phenotype (weakness, homonymous hemianopia and a focal cognitive deficit	Safety and tolerability	7 days

Brazil (Federal University of Rio de Janeiro)	2 arms (non randomized: 10 IA/5IV)	IV/IA	15	Autologous BMMCs	- MCA stroke - 18–75 yo - NIHSS 4 to 20	Safety	3 hrs to 90 days

IV: intravenous; IA: intra-arterial; IC: intracerebral; BMMC: bone marrow mononuclear cells; MCA: middle cerebral artery; NIHSS: National Institutes of Health Stroke Scale; TACS: total anterior circulation stroke.
